# *Babesia bovis* infection alters the composition and assembly of *Rhipicephalus microplus* midgut microbiota

**DOI:** 10.3389/fmicb.2025.1608409

**Published:** 2025-09-02

**Authors:** Iván Corona-Guerrero, Apolline Maitre, Lianet Abuin-Denis, Rodrigo Morales-García, Consuelo Almazán, Dasiel Obregón, Alejandro Cabezas-Cruz, Juan Mosqueda

**Affiliations:** ^1^Immunology and Vaccines Laboratory, C. A. Natural Sciences School, Autonomous University of Querétaro, Querétaro, Mexico; ^2^Doctorado en Ciencias Biológicas, Natural Sciences School, Autonomous University of Querétaro, Querétaro, Mexico; ^3^ANSES, INRAE, École Nationale Vétérinaire d’Alfort, UMR BIPAR, Laboratoire de Santé Animale, Maisons-Alfort, France; ^4^INRAE, UR 0045 Laboratoire de Recherches sur le Développement de l’Élevage (SELMET-LRDE), Corte, France; ^5^EA 7310, Laboratoire de Virologie, Université de Corse, Corte, France; ^6^Animal Biotechnology Department, Center for Genetic Engineering and Biotechnology, Havana, Cuba; ^7^School of Environmental Sciences, University of Guelph, Guelph, ON, Canada; ^8^C.A. Salud Animal y Microbiología Ambiental. Facultad de Ciencias Naturales, Universidad Autónoma de Querétaro, Querétaro, Mexico

**Keywords:** *Rhipicephalus microplus*, *Babesia bovis*, microbiota, network analysis, keystone bacteria

## Abstract

**Introduction:**

*Babesia bovis* is one of the main causative agents of bovine babesiosis. Livestock farmers are constantly struggling to control the population of the tick vector and reduce babesiosis outbreaks. For this reason, the development of new control strategies is necessary. Tick microbiota consists of a diverse group of symbiotic, commensal, and pathogenic microorganisms. It has been shown that altering the microbiota population prevents the transmission of apicomplexan pathogens. This work represents a primary exploratory approach to determine the changes *B. bovis* infection causes in the microbiota of *R. microplus*.

**Methods:**

Two calves were infested with *R. microplus* larvae; next, one of the calves was splenectomized and infected with Babesia bovis. Fifteen days after the infestation, engorged females were collected from each calf. Collected ticks were separated into two groups: 0 h and 72 h. Ticks from the 0 h group were dissected to extract their midgut the same day they were collected, while midgut dissection of the other group was done after 72 h of incubation. Thus, samples were separated into 4 experimental groups depending on their infection status and the time of the dissection. Total DNA was purified and the V4 region of the bacterial 16S rRNA gene was sequenced using Illumina MiSeq technology.

**Results:**

Data analysis showed fewer complex networks with reduced connectivity in infected ticks compared to the uninfected group. In both groups, the tick microbiota networks showed reduced node density at 72 h post-repletion. Different keystone taxa were found in all groups, indicating that midgut microbiota assembly is influenced by both tick developmental stage and the infection with *B. bovis*.

**Discussion:**

Results of this work aim to serve as a steppingstone in the development of anti-tick microbiota vaccines capable of impairing both the life cycle of *R. microplus* and *B. bovis* transmission.

## 1 Introduction

Ticks are obligate hematophagous ectoparasites that inhabit temperate, tropical, and subtropical regions worldwide (Sonenshine and Roe, 2014). These parasites are extremely prolific, capable of remaining in the environment and infesting multiple hosts, making them efficient vectors for pathogens (Estrada-Peña, 2015). While typically displaying tropism toward their main host, ticks can occasionally infest incidental hosts in severe starvation cases (Anderson and Magnarelli, 2008).

The ixodid tick, *Rhipicephalus microplus* inhabits tropical and subtropical regions around the world and has a host tropism toward cattle (Esteve-Gasent et al., 2020). Particularly, Mexico is a country where most of its territory has a suitable climate for the development of *R. microplus* and, thus, the tick is present in a large fraction of the national territory (Diario Oficial de la Federación, 1995). According to the status of the control of *R. microplus*, Mexican authorities have divided the country into three zones: the free zone (there is no natural presence of the tick), the eradication zone (systematic treatments and constant epidemiologic vigilance are carried out to eradicate the tick) and the control zone (epidemiologic surveillance and treatments are performed to lower the impact of the tick) (Diario Oficial de la Federación, 1995). *Rhipicephalus microplus* is present in the totality of the control and eradication zones, which consist of approximately 69.40% of the country’s territory (SENASICA, 2023).

The infestations caused by *R. microplus* can be extremely severe for livestock, causing economic losses due to weight loss, milk production decrease and damaged leather (Grisi et al., 2014). Ticks are also efficient vectors for pathogens due to their biology, ecology, and life cycle characteristics (Estrada-Peña, 2015). Tick prevalence prevents livestock farmers from introducing high-production breeds such as Holstein, Shorthorn or Hereford-Cebu due to their susceptibility to tick infestations in comparison to low production-high resistance breeds like Brahman and Brahman-crossbreeds (Jonsson et al., 2000; O’Kelly and Spiers, 1976). Effective tick control strategies are necessary to lower the population of the vector and reduce the incidence of tick-borne diseases such as bovine babesiosis.

*Babesia bovis* and *Babesia bigemina* are the most common causative agents of bovine babesiosis in Mexico (Alvarez et al., 2004). These apicomplexan parasites cause clinical manifestations such as acute fever, hemolytic anemia, hemoglobinuria, jaundice, anorexia, abortions, and weight loss and can result in the death of the animal (Bock et al., 2004). *Babesia bovis* causes aggregation of infected erythrocytes in lung and brain capillary blood vessels resulting in a higher mortality rate in susceptible adults (Brown and Palmer, 1999).

There are multiple strategies to control the tick population and *Babesia* infection. Acaricides have been universally used by livestock farmers for being cost-effective solutions to tick infestations, however, acaricides have important drawbacks such as safety concerns for non-human animals, soil and water accumulation, persistence in meat and selection of multi-resistant tick strains (Graf et al., 2004). These constraints made necessary the development of new integral strategies to control the tick population. An effective vaccine-centered strategy was developed by multiple groups, in which Bm86 of *R. microplus* (de la Fuente et al., 1999). Although the application of Bm86 vaccines was successful in reducing the use of acaricides to control tick infestations, its use has been limited due to the variability of Bm86 between tick strains (Merino et al., 2013).

Immune prophylaxis approaches have been employed to reduce the effect of *Babesia* on cattle. The first *Babesia* vaccines were made using live, attenuated parasites (Timms et al., 1983). Although live vaccines confer long-lasting protective immunity, they have short shelf life and the risk of reverting to a virulent phenotype (Timms et al., 1990). Recombinant vaccines, though safer, offer reduced long-term immune responses, carry a risk of reverting to *Babesia* pathogenicity, requiring cattle to be re-immunized to maintain protection against the pathogen (Jaramillo Ortiz et al., 2019; Jerzak et al., 2023).

A novel strategy employing vaccines that target the tick microbiota has been proposed. This approach is supported by evidence demonstrating that shifts in microbiota composition can impair vector feeding performance, offspring viability, and pathogen transmission capacity (Aželytė et al., 2022; Li et al., 2018; Mateos-Hernández et al., 2020; Narasimhan et al., 2017; Wu-Chuang et al., 2023). The objective of anti-tick microbiota vaccines is modulating the microbial community by immunizing the vertebrate host with a keystone taxon from microbiome to produce antibodies that, when ingested in the tick’s bloodmeal, will target specific taxon, disturbing the tick microbiota assembly and functionality (Mateos-Hernández et al., 2021).

Tick microbiota is comprised of diverse bacteria, fungi, archaea, protozoa, and viruses (Xia et al., 2015; X.-L. Zhang et al., 2022). Obligate symbionts like *Wolbachia spp.* are considered core microbiota, while microorganisms such as *Babesia* or *Anaplasma*, obtained through interactions with the environment are considered part of the facultative microbiota (Bonnet and Pollet, 2021; Nikoh et al., 2014). The microorganisms of the tick microbiota perform multiple functions such as synthesis of vitamin D and other cofactors to compensate for the lack of these nutrients in the blood meal (Duron et al., 2018; Moran and Baumann, 2000).

To pinpoint the keystone taxa in ticks, it is essential to comprehensively characterize their tissue-specific microbiota, including analyses of community composition, assembly processes, and functional interactions. During each life stage, multiple factors—including vertically transmitted symbionts, environmental exposures during questing, host skin microbiota, and acquired pathogens—dynamically reshape the microbial community (Garcia Guizzo et al., 2023). Notably, certain pathogens can induce dysbiosis within the vector, thereby enhancing their infectivity and survival (Tolker-Nielsen, 2015).

To date, no studies have compared changes in microbiota composition in response to *Babesia* infection in *R. microplus* ticks. Understanding the microbiota of *R. microplus* is essential for the development of anti-tick microbiota-based vaccines. The present work represents a first exploratory study aiming to characterize the microbiota composition of *R. microplus* at two stages of the *Babesia bovis* infection cycle and to assess how the parasite alters the tick’s microbial network. Data obtained from this study will serve as reference for the design of anti-tick microbiota vaccines.

## 2 Materials and methods

### 2.1 *Rhipicephalus microplus* infestation and *B. bovis* infection

Two European breed (*Bos taurus*) calves at least 6 months old and weighing less than 150 kg were selected for the experiment. The calves used for this experiment were obtained from the experimental ranch Valle del Guadiana in Durango, Mexico (part of the *R. microplus* control zone). Absence of anti-*Babesia* antibodies was confirmed by immunofluorescence antibody test (IFAT, not shown). Selected bovines were transferred to the Autonomous University of Queretaro (UAQ) Animal Infectomics Building and partially immobilized using a cattle chute to prevent the bovines from removing the ticks by grooming while allowing them to stretch, feed and lay down in non-stressing positions. Bovines had access to food and water *ad libitum*.

Because *Rhipicephalus microplus* is a one-host tick species, infestations in cattle must be initiated from the larval stage. Maintaining all the ticks in the same host removes the variable of facultative microbiota obtained from the interaction with different hosts and establishes each individual tick as a biological replica. Each calf was infested with 0.25 g of Media Joya strain *R. microplus* larvae (approx. 5,000 larvae). The larvae fed on the calves for 21–25 days, until they developed into fully engorged adult females. At 7 days post-infestation, one of the calves underwent a splenectomy via a left lateral approach at the Large Animal Veterinary Hospital of UAQ. Eight days after the splenectomy, it was infected with *Babesia bovis* via both intravenous and intramuscular routes, using two 2-ml vials containing 1 × 10^8^ infected erythrocytes (Hernández-Arvizu et al., 2024).

Clinical signs were monitored daily during the infection period (temperature, parasitemia, hematocrit, and other clinical signs related to *B. bovis* infection). Once the engorged ticks were collected from the infected calf, imidocarb dipropionate was administered at a dose of 3 mg/kg as therapeutic treatment for babesiosis. The Bioethics Committee of the Faculty of Natural Sciences at the Autonomous University of Querétaro (protocol number 053FCN2023) approved the non-human animal experimentation.

Starting at 21 days after the initial infestations, engorged female ticks were collected daily during a 5-day period. Collected ticks were transferred to the Immunology and Vaccines Research Laboratory (LINVAS) of the UAQ, Airport Campus to be processed. Ticks were washed by submersion in 10% benzalkonium chlorine for 5 min, rinsed with distilled water and dried with disposable paper towels. Half of the disinfected ticks were dissected the same day they were collected while the other half were incubated at 28°C and 80% relative humidity to be dissected after 72 h. At the end of the collection, ticks were separated into 4 groups: non-infected ticks dissected the same day of collection (0HN), non-infected ticks dissected 72 h after collection (72HN), infected ticks dissected the same day of collection (0HI), and infected ticks dissected 72 h after collection (72HI).

### 2.2 Tick dissection and DNA extraction

Ticks were fixed dorsally to petri dishes using double-sided tape. Ticks were opened by the posterior region of the body with a longitudinal cut to extract the midgut. Extracted tissues were washed using sterile phosphate-buffered saline (PBS, pH = 7.2) and stored individually in sterile PBS at −80°C. As a negative (blank) control for potential reagent or handling contamination, 500 μl of the same sterile PBS used for tissue rinsing was aliquoted, frozen, and carried through the entire DNA-extraction and sequencing workflow alongside the tick samples.

Tick organs were thawed in ice bath and centrifuged at 1000 × *g* for 30 s and the PBS was removed. Tick tissue was frozen using liquid nitrogen, then 180 μl of ATL buffer and 20 μl of proteinase K solution from the DNeasy Blood and Tissue Kit (QIAGEN, USA) were added. Immediately after, tissue was macerated using a sterile micro pestle. Following that, extraction was performed following instructions from the manufacturer. DNA extraction was also performed in negative control PBS to check for contamination. DNA integrity was verified by running an agarose gel electrophoresis. A NanoDrop 2000 was used to determine DNA concentration and purity.

### 2.3 Amplicon sequencing of bacterial 16S rRNA gene

DNA samples extracted from individual organs, with a 260/280 absorbance ratio ≥2.8 and concentration ≥10 ng/μl were sent to Novogene (Novogene Corporation Inc., Beijing, China). The V4 region of the 16S rRNA gene of bacteria and archaea was sequenced using the universal primers 505F/806R. All the samples were performed in the same batch. Analyses were performed on 7, 10, 8 and 9 samples from de 0HN, 72HN, 0HI and 72HI groups, respectively. These samples were sequenced using the pair-end approach in an Illumina MiSeq platform (Illumina, California, USA) service.

### 2.4 Bioinformatic analysis

The raw sequences were processed using the QIIME2 (version 2022.11) pipelines (Bolyen et al., 2019). Denoising, quality trimming, and merging processes were made using DADA2 method (Callahan et al., 2016). Taxonomic classifications of ASVs were done using a classify-sklearn naïve Bayes taxonomic classifier, which was built upon the SILVA database (release 138) (Quast et al., 2012). The taxonomic data table was examined at species level to corroborate that *Babesia* mitochondrial 16s rRNA genes were identified only in infected samples. Next, the taxonomic data table was aggregated at the genus level and used for the assessment of microbiome composition and network analysis.

### 2.5 Statistical analysis

Microbial diversity metrics were calculated and ASVs level. Alpha-diversity analyses were based on microbial richness (observed ASVs) and evenness (Pielow evenness) for the two-time points under both conditions (uninfected *vs.* infected). Differences in alpha-diversity metrics between groups were assessed with the pairwise Kruskal–Wallis test (*p* < 0.05) within QIIME2 (Bolyen et al., 2019). Beta diversity measures were based on Bray–Curtis dissimilarity index (Bray and Curtis, 1957). Comparison between groups were performed using PERMANOVA test (*p* < 0.05), while beta dispersion (intragroup dispersion) was calculated and compared using ANOVA test (*p* < 0.05), using the betadisper function and the Vegan script (Oksanen et al., 2022) implemented in R (R Core Team, 2023). Bray-Curtis’s dissimilarity was plotted using Principal Coordinate Analysis (PCoA; Gower, 2015). Then, we used Tukey’s Honest Significant Difference (HSD) Test to make pair-wised comparisons of beta-dispersion between groups (Tukey, 1949). To assess differences in taxonomic composition between groups, the taxonomic composition table at the genera level and filtered to exclude the rare taxa (<10 sequences, and prevalence <20%). The ALDEx2 R package was used to transform the raw number of reads into center-log ratio values. This allows us to quantify and compare each taxa abundance within and between samples while avoiding an overall bacterial load bias (Fernandes et al., 2013). Only taxa with significant differences (*p* < 0.05) were selected for representation on a heatmaps of the differential abundance taxa, created using the “Heatplus” package in R (Ploner, 2024).

### 2.6 Co-occurrence networks analyses

Co-occurrence networks were built with the read number values from the taxonomic table using the SparCC algorithm from the SpiecEasi package (Kurtz et al., 2015) in R (version 4.3.1) (R Core Team, 2023), using a SparCC cutoff of 0.75. Networks were visualized and processed using Gephi (version 0.10) (Bastian et al., 2009). Next, we used the NetCoMi approach to determine significant differences in network metrics among groups using the Jaccard Index test, built into the NetCoMi R package to compare degree, betweenness centrality (BC), closeness centrality, eigenvector centrality and hub taxa of the most central nodes of each network (Peschel et al., 2020).

#### 2.6.1 Network robustness

Robustness was evaluated by node removal to compare the network stability between different conditions. Network resistance to random and direct node removal was tested using the swan_combinatory function of the NetSwan package (Lhomme, 2015). This involved calculating the fraction of nodes that needed to be removed to cause a connectivity loss of 80%, following both directed (e.g., based on betweenness, cascading effects and degree centrality) and random removal strategies. Directed node removal was performed by removing the highest connected node (degree method), removing the node with higher BC (betweenness method), and removing the node with the higher BC value, then recalculating BC before removing the next (cascading method). The standard error for loss of connectivity was calculated, considering variability, using a threshold of 0.975, using bootstrapping methods to calculate confidence intervals.

#### 2.6.2 Determination of keystone taxa

To determine keystone taxa three criteria were employed: Firstly, these taxa had to be ubiquitous, signifying that were present across all samples within a particular group. Secondly, their eigenvector centrality had to be higher than 0.75 (Mateos-Hernández et al., 2021; Wu-Chuang et al., 2023). Eigenvector centrality is a metric for measuring the influence of a node within a network. Lastly, the mean relative abundance of these taxa needed to exceed that of the mean relative abundance of all taxa in the experimental group. To conduct this analysis, eigenvector centrality values were extracted from Gephi 0.10 software. All taxa were plotted on a two-axis graph using Graphpad Prism, indicating the cutoff points for CLR and Eigenvector centrality.

## 3 Results

### 3.1 Clinical signs of experimental calves

Starting the day of the infection, hematocrit, body temperature and parasitemia were checked daily. No abnormal clinical signs were observed on the uninfected calf. On the infected individual, no change on the clinical signs was observed from day 0 to day 5 post infection. On day 6, the first *Babesia bovis* trophozoite was detected in Giemsa-stained blood smears by light microscopy. Parasitemia was below 0.01% from day 0 to 7, increasing to 0.01% on day 8, to 0.2% on day 9 and to 4.60% on day 10. Body temperature remained at 38°C until day 7 post infection, increasing to 38.5°C on day 8, 40.6°C on day 9 and to 41.6°C on day 10. Hematocrit remained at 34% up until day 8, lowering to 27% on days 9 and 10 Imidocarb propionate was given to the infected calf on day 10 in response to acute clinical babesiosis.

### 3.2 Alpha and beta diversity metrics of R. microplus microbiota

Quality control results from Novogene showed that all samples passed the pre-amplification test, hence having the DNA concentration and purity necessary to carry out the Illumina sequencing process. Negative control samples showed no amplification, indicating no contamination was present.

To provide molecular confirmation of tick infection, we re-queried the 16S-amplicon dataset for apicomplexan sequences and detected *Babesia bovis* mitochondrial 16S rRNA fragments (≥93.1% identity to GenBank U06105.1) exclusively in the infected groups (0HI and 72HI; 116-146594 reads per sample) and absent from all uninfected samples. These parasite reads, removed prior to the bacterial network analysis as stipulated by our genus-level pipeline, are provided in Supplementary Table 1 together with their FASTA sequences.

**FIGURE 1:**
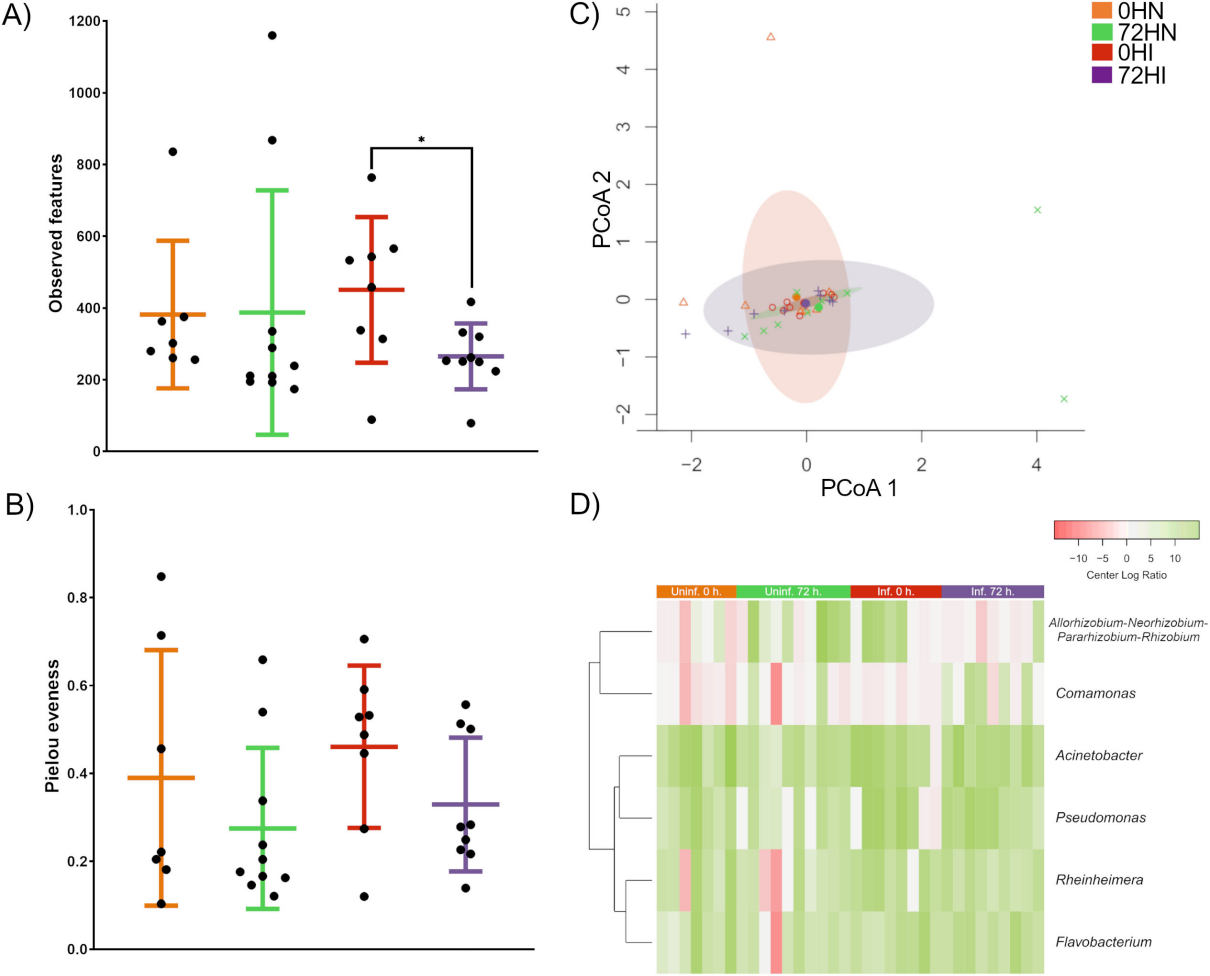
Analysis of alpha and beta diversity of midgut microbiota. **(A)** Comparison of taxa richness showed a significant difference between the OHI and 72Hl groups (Kruskall-Wallis, *p* ≤ 0.05), the rest of the groups showed no significant difference (Kruskall-Wallis, *p* ≥ 0.05). **(B)** Pairwise comparison of taxa evenness showed no significant difference between samples (Kruskall-Wallis, *p* ≥ 0.05). **(C)** PCoA plot of multivariate dispersion analysis showed that all groups shared a similar microbial community composition (Tukey’s HSD, *p* > 0.05). **(D)** Comparison of the relative abundance of taxa with significant differences between groups. Relative abundance data was transformed using CLR and expressed as a color gradient (Kruskall-Wallis, *p* < 0.05).

Pairwise comparison of alpha diversity metrics showed significant difference in ASVs richness between 0HI and 72HI groups (Kruskall-Wallis, *p* ≤ 0.05) (Figure 1A). No significant difference was observed between the rest of the groups (Kruskall-Wallis, *p* > 0.05). Pairwise comparison of taxa evenness showed no significant difference between groups (Kruskall-Wallis, *p* > 0.05) (Figure 1B). The microbial community composition showed no significant difference between groups according to Permanova (*F* = 99, *p* > 0.5). The pairwise comparison also showed no statistical difference between groups (Tukey’s HSD, *p* > 0.05) (Figure 1C).

### 3.3 Differential taxonomic composition

By examining the eukaryotic mitochondrial 16S rRNA genes identified during the taxonomic classification step, we detected *Babesia* mitochondrial reads. These reads were present in samples from the 0HI and 72HI groups but were absent in the 0HN and 72HN groups. These results confirm the *B. bovis* infection in the infected groups.

A total of 228 unique bacterial genera were detected among all groups. Of these, 149 were found in the 0HN group, 216 in 72HN, 82 in 0HI, and 129 in 72HI (Supplementary Table 2). Among all samples, 46 taxa were shared, including: *Bacteroides*, *Borrelia, Coxiella* and *Escherichia-Shigella* (Supplementary Table 2). When comparing uninfected samples, we found 142 taxa shared between the 0 and 72 h groups, while 8 taxa were unique to the 0HN and 75 were unique to 72HN. In the *B. bovis*-infected samples, 58 taxa were shared, with 25 taxa unique to the 0HI group and 71 unique to the 72HI group. We found 6 taxa with a significantly different relative abundances between groups (*Allorhizobium-Neorhizobium-Pararhizobium-Rhizobium, Comamonas, Acinetobacter, Pseudomonas, Rheinheimera and Flavobacterium)* (Kruskall-Wallis, *p* < 0.05) (Figure 1D).

### 3.4 Microbial co-occurrence network

**FIGURE 2:**
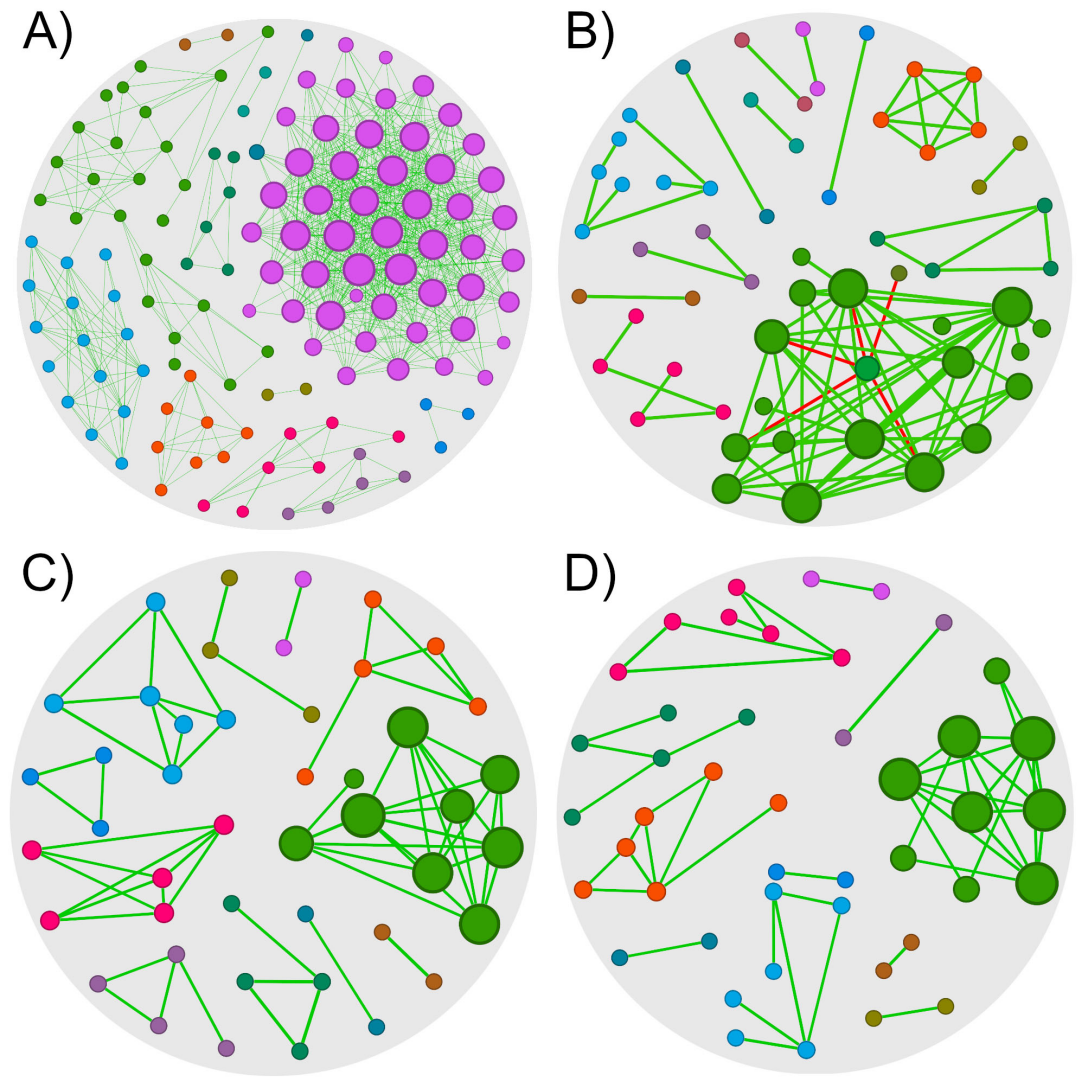
Co-occurrence networks of *R. microplus* midgut microbiota. Microbial co-occurrence networks of each group of samples (cutoff = 0.75). Nodes are colored according to the network module they belong to, and their size is proportional to its eigenvector centrality. Edges are colored according to their type of correlation (green for positive correlations and red to negative correlations). **(A)** Network of the 0HN group, composed of 128 nodes with 753 edges (100% positive correlations). **(B)** Network of the 72HN group, composed of 58 nodes with 90 edges (94.4% positive correlations). **(C)** Network of the 0HI group, composed of 45 nodes with 64 edges (100% positive correlations). **(D)** Network of the 72HI group, composed of 44 nodes with 51 edges (100% positive correlations).

**FIGURE 3:**
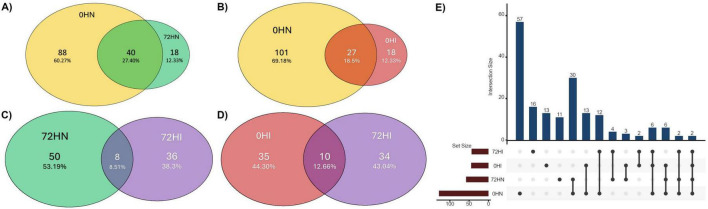
Venn diagram indicating the percentage of shared taxa between the 0HN (yellow), 72HN (green), 0HI (red) and 72HI (purple) networks. **(A)** Out of 146 total taxa, 0HN and 72HN share 40, 88 are unique to 0HN and 18 to 72HN. **(B)** Out of 146 total taxa, 0HN and 0HI share 27, 101 are unique to 0HN and 18 to 0HI. **(C)** The 72HN and 72HI groups have 94 taxa between both, sharing 8, 50 being unique to 72HN and 36 to 72HI. **(D)** The 0HI and 72HI groups have a total of 79 taxa, sharing 10 of them, while 35 are unique to 0HI and 34 to 72HI. **(E)** UpSet plot showing taxa shared among the 4 groups.

The 0HN, 72HN, 0HI, and 72HI networks had 128/753, 58/90, 45/64, and 44/51 nodes/edges, respectively (Figure 2 and Table 1). Both 0h networks and 72HI network had 100% positive correlations, while the 72HN network had 5 negative correlations (5.6%). In the four networks, a total of 220 unique taxa were found (Supplementary Table 3). From those, 57, 11, 13 and 60 were unique to the 0HN, 72HN, 0HI and 72HI groups, respectively. At the same time, only 2 taxa were shared among all 4 groups (Figure 3).

**TABLE 1:** Topological features of the co-occurrence networks. A comparative analysis of the topological features is presented comparing metrics for each of the networks from the 0HN, 72HN, 0HI, and 72HI groups.

Topological features	0HN	72HN	0HI	72HI
Nodes	128	58	45	44
Edges	753	90	64	51
Positives	753 (100%)	85 (94.4%)	64 (100%)	51 (100%)
Negatives	0 (0%)	5 (5.6%)	0 (0%)	0 (0%)
Modularity	0.402	0.659	0.809	0.771
Clustering coefficient	0.719	0.617	0.759	0.624
Network diameter	10	4	3	4

The 0HN exhibited the highest taxa richness and connectivity. Reduced richness and connectivity in 72HN, 0HI, and 72HI aligned with increased modularity. Modularity values were 0.402 (0HN), 0.659 (72HN), 0.809 (0HI), and 0.771 (72HI), indicating increasing modular partitioning. Additionally, networks had a clustering coefficient (CC) higher than 0.6 (0.719, 0.617, 0.759, 0.624 for 0HN, 72HN, 0HI and 72HI, respectively). Lower CCs in 72HN and 72HI suggest reduced interconnectivity over time post-repletion (Table 1).

### 3.5 Network topology comparison

**FIGURE 4:**
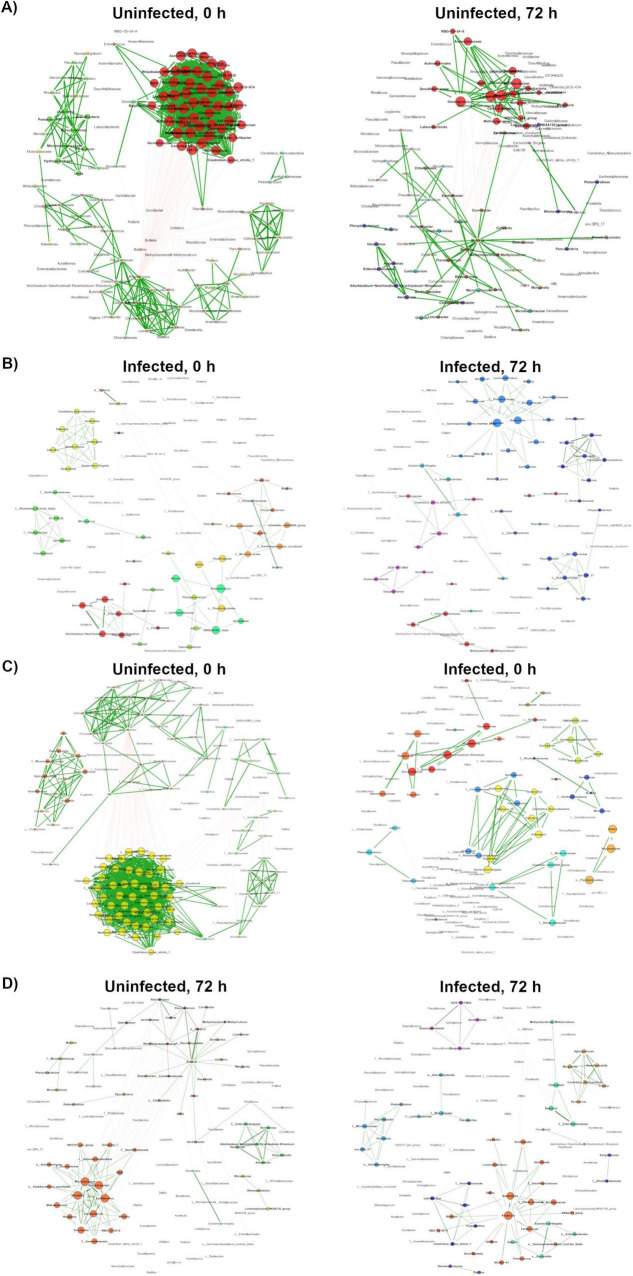
Network topology comparison using NetCoMi. Network topology comparison using the NetCoMi R package. In each comparison each dot represents a taxon, and taxa are located at the same position. **(A)** Comparison between OHN and 72HN networks. **(B)** Comparison between 0HI and 72HI networks. **(C)** Comparison between 0HN and 0HI networks. **(D)** Comparison between 72HN and 72HI networks.

The NetCoMi R package was used to compare the composition and topology of networks. Topology comparison between 0HN and 72HN showed a large taxa hub present in the first network, which remains after 72 h (Figure 4A). The remaining modules of both networks have different composition and topology. Comparing the 0HI with the 72HI we observed several taxa hubs, however, these hubs were composed of different taxa in each network (Figure 4B). Comparing the networks from uninfected and infected groups at 0 h post-repletion we can see that the large taxa hub in the 0HN network is not present in the 0HI group, additionally, the rest of the taxa clusters in the 0HN network are bigger than the ones in 0HI and have different components (Figure 4C). Each of the 72HN and 72HI networks have isolated taxa clusters with different compositions, indicating a different microbial community composition (Figure 4D). Results of the Jaccard Index tests showed a significant difference in the composition of the most central nodes of both uninfected (Table 2) and infected (Table 3) networks at 0 versus 72 post-repletion hours and that this difference was a product of the composition *per se* (*P*(< = Jacc)) and not randomness (*P*(> = Jacc)).

**TABLE 2:** Comparing 0HN and 72HN networks topological features using the Jaccard index test. Comparison between network metrics of networks from uninfected midgut samples at 0- and 72-h post-repletion.

Uninfected 0 h vs. 72 h	Jaccard index	*P* (≤Jacc)	*P* (≥Jacc)
Degree	0.247	0.065426	0.961308
Betweenness centrality	0.214	0.036709*	0.982061
Closeness centrality	0.188	0.001148**	0.999501
Eigenvector centrality	0.163	0.000127***	0.999953
Hub taxa	0.163	0.000127***	0.999953

*p < 0.05,

**p < 0.005,

***p < 0.005.

**TABLE 3:** Comparing 0HI and 72HI networks topological features using the Jaccard index test. Comparison between network metrics of networks from infected midgut samples at 0- and 72-h post-repletion.

Infected 0 h vs. 72 h	Jaccard index	*P* (≤Jacc)	*P* (≥Jacc)
Degree	0.247	0.065426	0.961308
Betweenness centrality	0.214	0.036709*	0.982061
Closeness centrality	0.188	0.001148**	0.999501
Eigenvector centrality	0.163	0.000127***	0.999953
Hub taxa	0.163	0.000127***	0.999953

*p < 0.05,

**p < 0.005,

***p < 0.005.

### 3.6 Changes of network robustness

**FIGURE 5:**
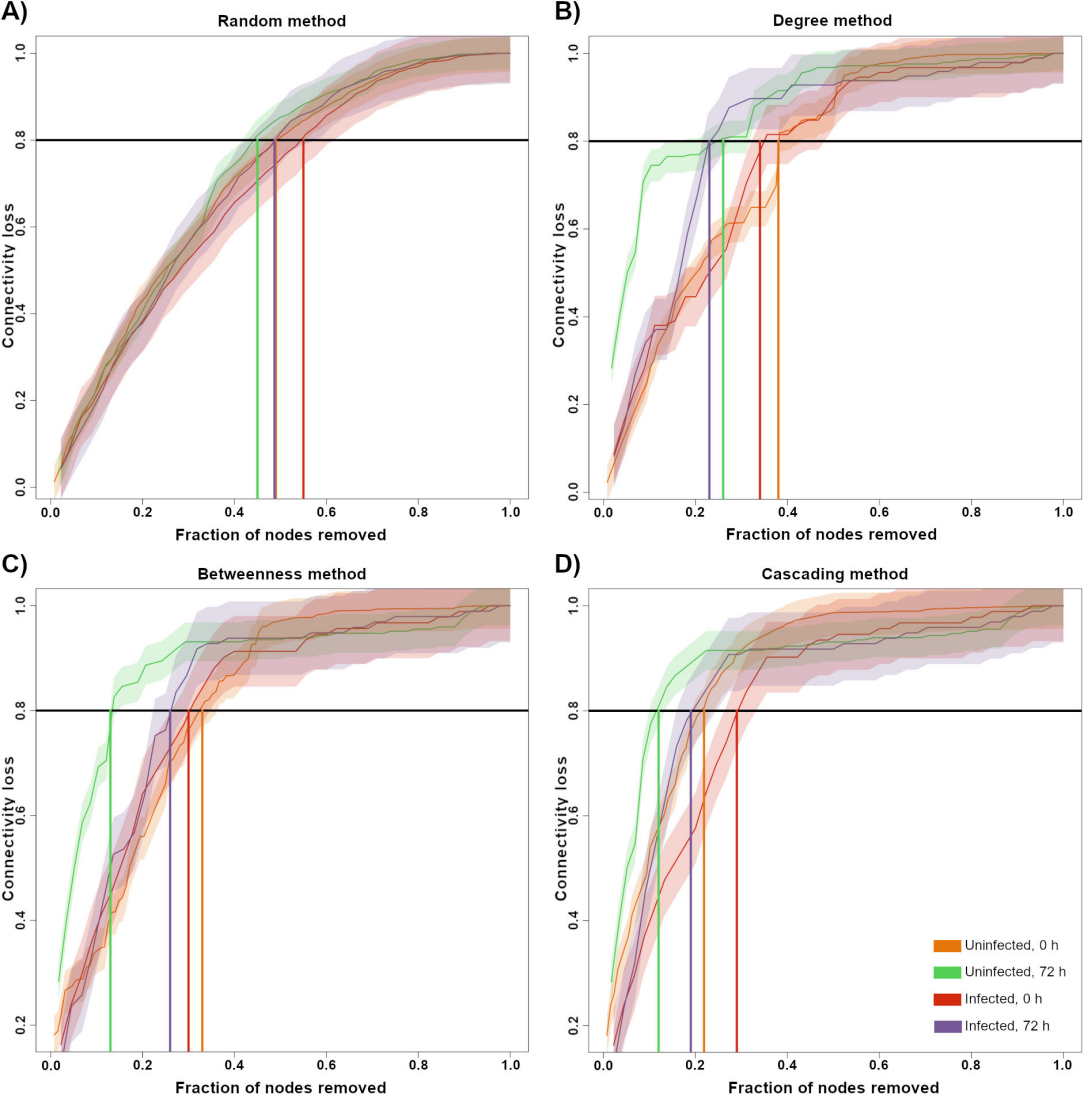
Robustness test by node removal. A network vulnerability test by node removal was conducted for the 0HN (orange), 72HN (green), 0HI (red) and 72HI (purple) networks using four different methods. Nodes were removed using at random **(A)**, by higher degree **(B)**, by higher betweenness centrality **(C)** and using a cascading method **(D)**. En each graph, the *X*-axis represents the fraction of nodes that were removed from the network and the *Y*-axis represents the fraction of connectivity that was lost in response to that removal. The solid-colored line represents the relationship between the fraction of removed nodes of each sample with the connectivity loss and the shaded area is the standard error. The horizontal black line shows 0.8 of the total connectivity and vertical-colored lines shows the fraction of nodes removed at which the network loses 0.8 of the connectivity, color code is presented in the bottom right.

Robustness was evaluated by calculating the percentage of connectivity loss after removing nodes. Randomly removing nodes from the network showed no difference in the connectivity loss in any group (Figure 5A). By removing higher degree nodes first, we observed that 72HN group lost the most connectivity with the least fraction of nodes removed, followed by the 72HI group. 0HN and 0HI showed no noticeable difference when removing nodes by higher degree (Figure 5B). Removing nodes with the highest BC resulted in the 72HN losing connectivity with less fraction of node removal than the rest of groups. 0HN, 0HI and 72HI groups showed no difference among each other (Figure 5C). The cascading method showed that 72HN group was the least robust, followed by 0HN and 72HI and lastly 0HI (Figure 5D).

### 3.7 Identification of keystone taxa

**FIGURE 6:**
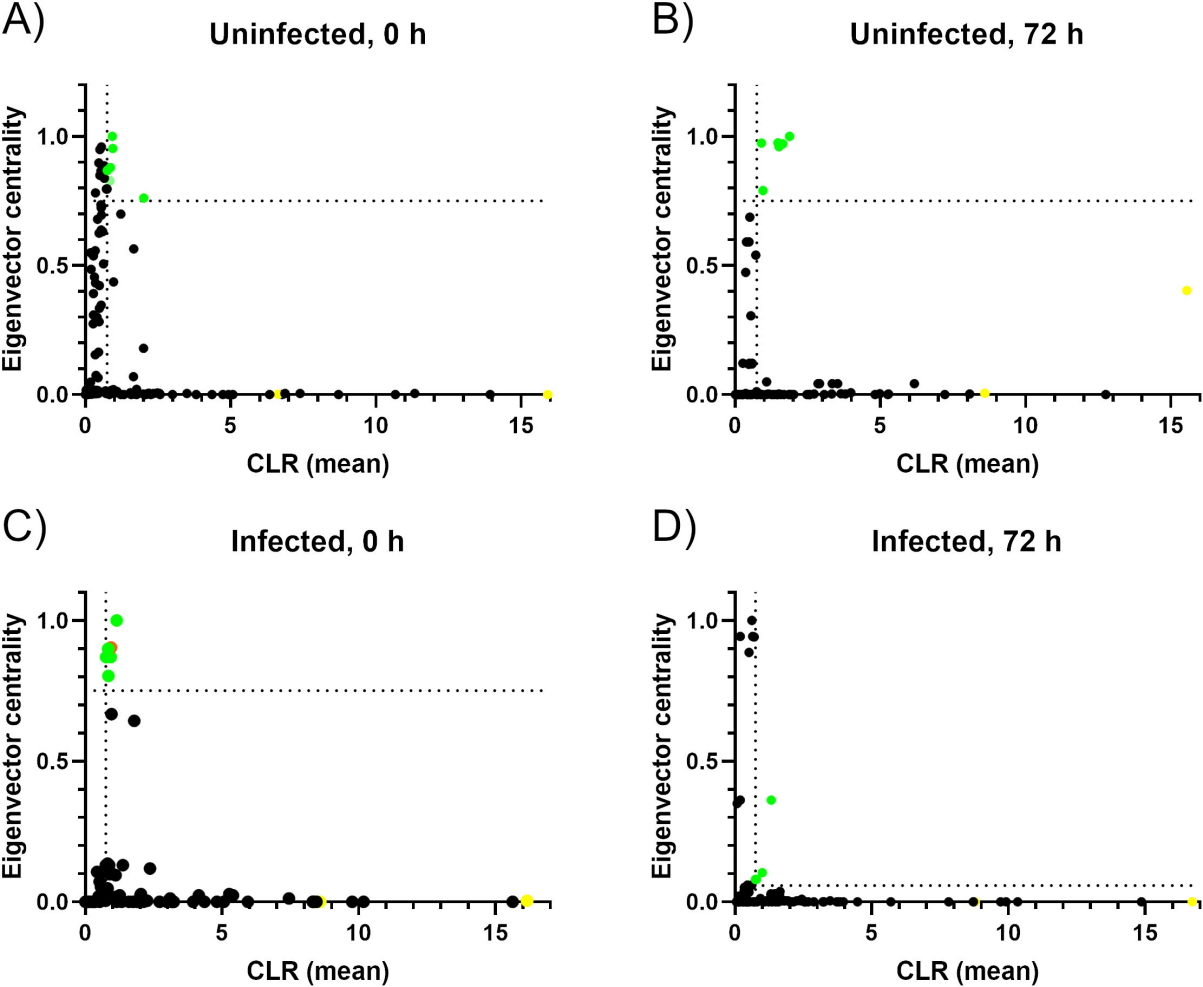
Keystone taxa determination based on relative abundance and connectivity. Graphs representing the keystone taxa of the 0HN **(A)**, 72HN **(B)**, 0HI **(C)** and 72HI **(D)**. Each graph represents the relative abundance as CLR-transformed values in the *X*-axis. The connectivity of each taxon is represented in the *Y*-axis with the eigenvector centrality value. Each taxon is represented as a dot. The vertical dotted line represents the mean of the CLR-transformed abundance data, and the horizontal dotted line represents the empiric 0.75 cutoff of the eigenvector centrality value **(A–C)** or the mean of the eigenvector centrality value **(D)**. A dot was considered as keystone taxa if it was above both cutoffs. The previously reported *Escherichia-Shigella* cluster (Mateos-Hernández et al., 2021) was colored in orange in the 0HI graph **(C)**. Dots corresponding to the known tick endosymbionts, *Borrelia* and *Coxiella*, were colored yellow in all graphs.

The 0HN group had 6 keystone taxa: Xanthobacteraceae (uncultured) (family), *Lactobacillus, Moraxella*, Acidobacteria subgroup 2 (phylum), *Xanthomonas* and Elsterales (order) (Figure 6A and Table 4). The 6 keystone taxa found in the 72HN group were: *Cetobacterium, Dolosigranulum, Lactobacillus*, *Moraxella*, *Muribaculaceae and Turicibacter* (Figure 6B and Table 4). The keystone taxa found in the 0HI group were the following: *Acidibacter, Anaerococcus, Candidatus Nomurabacteria*, *Escherichia-Shigella*, *Haliangium*, and *Ralstonia* (Figure 6C and Table 4). Lastly, keystone taxa found in the 72HI were: Planococcaceae (family), *Bacteroides*, *Koukoulia*, Burkholderiales SC-I-84 (order) (Figure 6D and Table 4).

**TABLE 4:** Keystone taxa found for each co-occurrence network.

Keystone taxa (0HN)	CLR	EVC
Xanthobacteraceae (uncultured) (f)	0.842	0.828
*Lactobacillus*	2.016	0.761
*Moraxella*	0.943	0.953
Acidobacteria (p) subgroup 2	0.929	1.000
*Xanthomonas*	0.758	0.869
Elsterales (o)	0.864	0.880
**Keystone Taxa (72HN)**	**CLR**	**EVC**
*Cetobacterium*	0.965	0.790
*Dolosigranulum*	1.476	0.976
*Lactobacillus*	1.882	1.000
*Moraxella*	1.646	0.971
*Muribaculaceae*	1.515	0.961
*Turicibacter*	0.910	0.974
**Keystone taxa (0HI)**	**CLR**	**EVC**
*Acidibacter*	1.149	1.000
*Anaerococcus*	0.761	0.869
*Candidatus Nomurabacteria*	0.928	0.869
*Escherichia*-Shigella*	0.936	0.903
*Haliangium*	0.845	0.802
*Ralstonia*	0.834	0.899
**Keystone taxa (7HI)**	**CLR**	**EVC**
*Planococcaceae* (f)	0.753	0.080
*Bacteroides*	0.792	0.080
*Koukoulia*	0.994	0.104
Burkholderiales (o) SC-I-84	1.330	0.361

*Previously reported as Keystone taxa by Mateos-Hernández et al. (2021)

## 4 Discussion

It has previously been reported that colonization by pathogenic microorganisms can alter the tick microbiota composition during the infection process (Maitre et al., 2022; Narasimhan et al., 2017). Our findings support the hypothesis that microbiota from *R. microplus* differs between uninfected ticks and ticks infected with *B. bovis*. Additionally, the microbiota composition in both uninfected and uninfected ticks changed significantly between 0 and 72 h after engorgement. Understanding the shifts in microbiota composition between these two time points is crucial for elucidating its role in *Babesia* infections. *B. bovis* invasion of the tick midgut begins when merozoites are ingested by ticks at 0 h post-repletion. Inside the tick, these merozoites develop into sexual stages (gametocytes), which subsequently transform into infective forms that penetrate the midgut epithelium. By 72 h post-repletion, these forms differentiate into kinetes, a stage coinciding with cellular effects such as the upregulation and re localization of the voltage-dependent anion channel (BmVDAC) in *R. microplus* midgut cells (Rodríguez-Hernández et al., 2012, 2015).

Tick susceptibility to pathogen infection is mediated by multiple mechanisms. First, humoral components of the tick immune system like anti-microbial peptides are capable of binding to the parasite surface and inhibit their proliferation (Tsuji et al., 2007; Yao et al., 2021). Additionally, it has been demonstrated that microbiota composition can alter multiple arthropod susceptibility to pathogen infection in ixodid models transmitting pathogens like *Rickettsia helvetica or* (Gall et al., 2016; Maitre et al., 2022), *Anaplasma phagocytophilum* (Abraham et al., 2017) and *Babesia microti* (Wei et al., 2021).

### 4.1 Decrease in microbiota diversity

During the feeding process of ixodid ticks, the midgut lumen fills with the blood meal and functions as a reservoir, separated from the midgut epithelium by the peritrophic matrix (PM; Sonenshine and Roe, 2014). Nutrients and cellular debris can selectively cross the PM and bind to membrane receptors of epithelial cells to initiate receptor-mediated endocytosis. Once internalized, the nutrients are digested intracellularly and transported to the basal lamina. This process is taxing for the epithelial tissue, which must constantly replace damaged cells, ultimately leading to tissue degradation at the onset of oviposition (Sonenshine and Roe, 2014).

Our analytical pipeline—centered log-ratio normalization with ALDEx2 followed by differential-abundance testing— was intentionally chosen to fulfill the study’s primary aim: to capture the emergent, community-level effects of *Babesia bovis* infection on the *Rhipicephalus microplus* mid-gut microbiota, rather than to track quantitative shifts of individual bacterial taxa. These methods provide robust relative-abundance data that underpin the network-level reconfiguration reported here. At the same time, we recognize the complementary value of absolute-quantification approaches such as taxon-specific qPCR or droplet-digital PCR. Future investigations focusing on the mechanistic roles of the differentially abundant or keystone genera identified in this study could employ these techniques to validate and extend the hypotheses generated by our community-scale analysis.

Alpha diversity metrics showed no significant differences in richness or evenness of taxa between samples, except for a difference in richness between the 72HI and 72HN groups. A PCoA analysis indicated a similar taxonomic composition among groups. These results suggest a comparable number of individual reads across samples, with a uniform proportion of taxa. However, co-occurrence network analysis revealed a significant decrease in both the number and connectivity of nodes in the 0HI group compared to the 0HN group. When comparing the 72-h and 0-h groups, we observed a decrease in the number of nodes in the microbial networks of uninfected individuals; however, this decrease in diversity was not observed in the infected groups. These findings indicate that *B. bovis* infection has a direct impact on the microbiota diversity at 0 h-post repletion. Similar results have been previously reported in *R. microplus* where the infection with *Theileria sp.* correlates with a loss of bacterial diversity and richness on the vector’s tissues (Adegoke et al., 2020). Another tick model showing a similar response is *Ixodes scapularis*, where infection with *Anaplasma phagocytophilum* alters the microbiota composition (Abraham et al., 2017). Microbiota alterations caused by pathogens have also been observed in other vectors sucha as *Culex quinquefasciatus* where *Plasmodium relictum* infection decreases the diversity of the midgut microbial community (Aželytė et al., 2022). Different symbionts and pathogenic bacteria colonize specific tissues depending on their roles in the microbial community and the tick’s life stage (Wiesinger et al., 2023; X.-Y. Zhang et al., 2022). These community dynamics might explain the decrease in diversity observed at 72 h post-repletion rather than being solely attributable to *B. bovis* infection.

### 4.2 Comparison of co-occurrence networks

The 0HN network had the highest node count of all the experimental groups. When comparing this group to 72HN, we can see that the number of nodes and edges decrease. This loss of connectivity can be attributed to the midgut degradation (Sonenshine and Roe, 2014). Additionally, a similar decrease in these topology features can be observed when comparing the 0HN to the 0HI group. Microbial networks from these two groups have a similar number of nodes and edges, and the shared taxa are highly dissimilar (sharing only 12.66% of their total taxa). This phenomenon can be attributed to damage to the PM by the *Babesia* arrowhead structure-contained enzymes (Jalovecka et al., 2018) or as collateral damage caused by non-specific innate immune response from the tick (Sonenshine and Hynes, 2008).

Seeing the significant differences in network composition in all groups, it became of interest to determine the robustness of the networks. The node removal robustness test showed that 0HN network retained more connectivity when removing a higher number of nodes compared with other networks. This could be explained by its more diverse community with complex interactions. It has been mentioned that microbial networks with these characteristics tend to be more resistant to connectivity loss in response to perturbations on the network composition (Konopka et al., 2015). This resistance to change referrers to compositional stability, not functional. We still need to determine if the difference in composition also implies a change in the metabolic function of the network.

Although we complemented SparCC-derived co-occurrence networks with robustness testing (NetSwan) and node-composition statistics (NetCoMi), we recognize that network topology can vary with the choice of inference algorithm, correlation threshold and compositional correction. A systematic sensitivity analysis—e.g., recalculating networks across a range of SparCC cut-offs and comparing results with alternative approaches such as SPIEC-EASI, CoNet, CCLasso or FlashWeave—was beyond the scope of the present work but is essential to gauge the stability of keystone-taxon identification and global metrics (modularity, clustering coefficient, eigenvector centrality). Cross-method validation and threshold testing are therefore critical next steps for consolidating community-level patterns and verifying that taxa highlighted as potential targets for anti-microbiota interventions are consistently recovered across analytical frameworks.

### 4.3 Comparison of keystone taxa

A single host can have different stable microbiota compositions. The assembly of each composition is influenced by environmental and dietary factors (Shetty et al., 2017). For each stable composition, keystone taxa can exert important influence in the microbiome function (Banerjee et al., 2018). Although previously reported symbionts of *R. microplus* such as *Coxiella* and *Borrelia* (Garcia-Guizzo et al., 2022; LaRocca et al., 2009) are present in all samples’ microbial communities, their lack of connectivity in the network assembly rules them out of the keystone taxa status. Uninfected groups share *Lactobacillus* and *Moraxella* as keystone taxa at both 0 and 72-h post repletion. The exact role that *Lactobacillus spp.* plays in the microbiome of ticks has not been determined, however, it has been deemed as a probiotic for the mulberry silkworm, *Bombyx mori* (Yeruva et al., 2020). Additionally, it has been shown to be highly abundant on the midgut microbiota of the soft tick *Ornithodoros moubata*, which made it an effective anti-microbiota vaccine target (Cano-Argüelles et al., 2024). *Moraxella spp.* has been associated with lipid metabolism in insects (Li et al., 2021). Lipid metabolism is necessary for vitellogenesis in insects and ticks during ovary maturation (Amaral Xavier et al., 2018; Tufail and Takeda, 2008), which makes this keystone taxa a potential vaccine candidate.

Infected groups don’t share any keystone taxa between the 0 and 72-post repletion hours. However, the *Escherichia-Shigella* cluster (ESC) was present in the 0HI group as a keystone taxon. Although works on *B. mori* suggest the ESC could be involved in the degradation of carbohydrates, the exact role of this taxa hasn’t been determined (Zheng et al., 2020). However, it has been previously used as an anti-microbiota vaccine antigen capable of altering the microbial community, potentially reducing metabolic pathways such as lysine degradation (Mateos-Hernández et al., 2021). The use of ESC as an anti-tick microbiota antigen has efficiently reduced the load of the pathogenic bacterium *Borrelia afzelii* in the vector (Wu-Chuang et al., 2023).

Other keystone taxa found in different groups might have different functions. For example, *Xanthomonas* was determined as a keystone taxon in the 0HN group. Bovine used for this experiment were obtained from field condition and plant-associated microbiota was most likely obtained from its interaction with the bovine host skin. This is what would be expected, since components from the host microbiota are often found in the vector midgut (Choubdar et al., 2021). We found *Muribaculaceae* among the keystone taxa of the 72HN group. This taxon was previously found as keystone taxa of the salivary glands’ microbiota of soft ticks, although its role on the microbiota it’s not yet known (Piloto-Sardiñas et al., 2023). *Turicibacter* is another keystone taxa found in the 72HI samples, its role in the tick’s midgut microbial community hasn’t been explored. However, in the stag beetle *Dorcus hopei hopei* model, *Turicibacter* is highly abundant in the gut microbiota of the larval stages and plays a role in energy metabolism and its positively related to higher growth (Lu et al., 2024).

We found *Anaerococcus* as a keystone taxon in the 0HI group. This bacterium is associated with short chain fatty acid production and promotion of anti-inflammatory state in mucous tissue of *Aedes albopictus* (Wei et al., 2023), although its role within tick microbiota is not known. *Acidibacter*, another keystone taxa found in the 0HI group, has been previously reported as a high abundance bacterium in *Haemaphysalis longicornis* (Zhang et al., 2023). Other taxa in the infected groups like *Haliangium, Ralstonia, Bacteroides* and *Koukoulia* have previously been reported in the microbiota of ticks and other arthropods (Keskin et al., 2017; Wei et al., 2013), they haven’t been reported as being highly prevalent or having a key role in their host’s metabolism (Keskin et al., 2017; Wei et al., 2013).

It has been previously reported that different bacteria species of the tick microbiota are correlated to their host’s susceptibility or resistance to tick borne pathogen infections (Gall et al., 2016). This effect can be achieved by manipulating their host gene expression. For example: *Anaplasma phagocytophilum* can induce the expression of the *Ixodes scapularis* anti-freeze glycoprotein (*iafgp)* gene, which thins the PM enabling the invasion of the pathogen (Abraham et al., 2017). It is important to take in count the roles of the keystone taxa when selecting the bacteria that will be used as an antigen in anti-tick microbiota vaccines. We must make sure the antigen candidates are positively correlated to tick fecundity and pathogen susceptibility. Additionally, we must determine that no other taxa in the network have redundant functions to effectively disrupt the microbiota composition (Konopka et al., 2015).

### 4.4 Implications in vaccine design

As mentioned previously, the removal of most central nodes from a network result in a quicker connection loss. Due to keystone taxa being the more central and abundant members of the microbial community, targeting those with an anti-microbiota vaccine is a specific and efficient way to remove these bacteria from the midgut microbiota (Mateos-Hernández et al., 2021). Previous studies showed that removal of one keystone species using vaccination strategies can induce major changes in the overall assembly of the microbial community (Aželytė et al., 2022). For these reasons it is important to study the composition of the tick microbiota and determine their keystone taxa to adequately design vaccines. Immunization against keystone taxa is necessary to cause a major disruption in the assembly of the tick midgut microbial community.

The present work also took into consideration the microbial community composition shift caused by the infection with *B. bovis*. The observed change in overall network composition and differential keystone taxa enables us to choose a keystone bacterium that, being used as a vaccine target, could both impair the *B. bovis* infection on the tick to stop its propagation and cause a dysbiosis state in the tick that hiders its biological fitness.

### 4.5 Most viable vaccine candidates

From the keystone taxa we identified, there are several we’d like to highlight as the most viable vaccine candidates. *Lactobacillus sp*. is a promising candidate which has been previously tested as a vaccine antigen due to being associated with bovine lactic acidosis (Shu et al., 1999). Immunized bovines produced good antibody titters and developed no adverse reactions in their rumen. However, our approach didn’t specify the *Lactobacillus* species present in the midgut microbiota, so it would be necessary to isolate and identify it. Isolation of *Lactobacillus* spp. requires the use of special culture media and anaerobic conditions, which further complicates the isolation and growth of this genera.

*Moraxella* spp. is another viable vaccine candidate. Vaccination against *Moraxella bovis* has been proven effective to prevent bovine keratoconjunctivitis-causing infections (Angelos et al., 2021). Same as with *Lactobacillus* spp., it is necessary to determine the species of *Moraxella* present in the tick midgut. *Moraxella* spp. can grow under standard conditions (Baumann et al., 1968), which could facilitate the migration to large scale vaccine production if necessary.

We can also consider the ESC as a potential vaccine candidate. It’s possible to use only one of the cluster’s bacteria to make a vaccine. *E. coli* can grow under permissive conditions and there are multiple commercially available strains. The use of *E. coli* in experimental vaccines have been proven effective to disrupt microbiota assembly and pathogen colonization in ticks (Mateos-Hernández et al., 2020; Wu-Chuang et al., 2023). In this case, the challenge of identifying the exact strain present in the tick midgut remains.

## 5 Limitations

In this exploratory work, we were able to determine composition and keystone taxa of the *R. microplus* midgut microbiota. Nonetheless, our approach presents limitations that open opportunity areas for further projects. One of such limitations is that this study focused on two milestones of the blood-digestion cycle—immediately after engorgement (0 h) and the late digestive phase (72 h)—because they bracket the window in which *B. bovis* parasites are first ingested and the time when they leave the midgut cells to infect other tissues. Nevertheless, microbiota composition can fluctuate markedly during the intervening hours as the PM forms, hemoglobin catabolism progresses, and epithelial turnover accelerates. By only sampling the start and end of this interval we may have missed short-lived taxa, transient interactions, or tipping points that precede the network re-configuration reported here. Future work that couples denser time-series sampling (e.g., 6, 12, 24, 48, 72 h) with absolute-quantification methods will be essential to reconstruct these intermediate transitions, clarify the chronology of keystone-taxon emergence, and determine whether the community-level patterns identified at 72 h persist, re-equilibrate, or diverge during the oviposition period.

By knowing the composition of the midgut microbiota, it’s possible to make a prediction of the metabolic profile of the bacterial assembly. By comparing the taxa present in the microbial networks and their relative abundance from data obtained through Illumina sequencing and comparing it to databases we can determine the metabolic capabilities of the microbiota. Metabolic prediction was outside the scope of this project, additionally, only the 16s rRNA marker was used, which could limit the metabolic prediction. A more comprehensive metabolic profile prediction would require the use of multiple molecular markers to include fungi, viruses and protozoa.

## 6 Conclusion

From an epidemiological standpoint, *B. bovis* is a difficult pathogen to control. It is prevalent in areas with high cattle density, young calves are resistant to infection and serve as reservoirs, and vectors such as *R. microplus* can persist in the field for prolonged periods (Jerzak et al., 2023; Spickler, 2018). Understanding the effects that *B. bovis* induces in the tick microbiota is a necessary step toward elucidating the vector–microbiota–pathogen interactions that underpin *R. microplus* vectorial capacity. In the present study, we investigated differences in midgut microbiota composition in engorged *R. microplus* females at various post-repletion time points and under different *B. bovis* infection statuses. The results indicate that *B. bovis* infection alters the structure and reduces the bacterial diversity of the *R. microplus* midgut microbiota at 0 h post-repletion. Notably, taxa associated with energy metabolism, such as *Moraxella* and *Turicibacter*, were absent in infected samples. Among the keystone taxa identified in different groups, we found bacteria previously reported as keystone species and as targets for anti-microbiota vaccines in other tick species, including *Lactobacillus*, *Moraxella*, *Muribaculaceae*, and *Escherichia-Shigella*. This information can be leveraged to advance the development of anti-tick microbiota vaccines. We hypothesize that using keystone taxa from both infected and uninfected midgut microbiota as antigens represents an optimal vaccine-based tick control strategy, potentially reducing *R. microplus* fitness and preventing the transmission of *B. bovis*.

## Data Availability

The status of the bioproject page was changed to released and a new public URL was provided: https://www.ncbi.nlm.nih.gov/bioproject/PRJNA1216386.
